# Learning to Identify Physiological and Adventitious Metal-Binding Sites in the Three-Dimensional Structures of Proteins by Following the Hints of a Deep Neural Network

**DOI:** 10.1021/acs.jcim.2c00522

**Published:** 2022-06-09

**Authors:** Vincenzo Laveglia, Andrea Giachetti, Davide Sala, Claudia Andreini, Antonio Rosato

**Affiliations:** † Consorzio Interuniversitario di Risonanze Magnetiche di Metallo Proteine, Via Luigi Sacconi 6, 50019 Sesto Fiorentino, Italy; ‡ Institute for Drug Discovery, Leipzig University, Brüderstr. 34, 04103 Leipzig, Germany; § Magnetic Resonance Center (CERM), University of Florence, Via Luigi Sacconi 6, 50019 Sesto Fiorentino, Italy; ∥ Department of Chemistry, University of Florence, Via della Lastruccia 3, 50019 Sesto Fiorentino, Italy

## Abstract

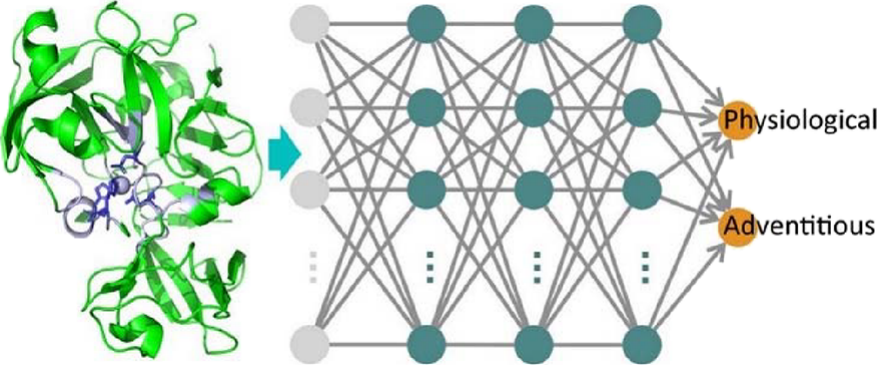

Thirty-eight percent of protein structures in the Protein Data Bank contain at least one metal ion. However, not all these metal sites are biologically relevant. Cations present as impurities during sample preparation or in the crystallization buffer can cause the formation of protein–metal complexes that do not exist in vivo. We implemented a deep learning approach to build a classifier able to distinguish between physiological and adventitious zinc-binding sites in the 3D structures of metalloproteins. We trained the classifier using manually annotated sites extracted from the MetalPDB database. Using a 10-fold cross validation procedure, the classifier achieved an accuracy of about 90%. The same neural classifier could predict the physiological relevance of non-heme mononuclear iron sites with an accuracy of nearly 80%, suggesting that the rules learned on zinc sites have general relevance. By quantifying the relative importance of the features describing the input zinc sites from the network perspective and by analyzing the characteristics of the MetalPDB datasets, we inferred some common principles. Physiological sites present a low solvent accessibility of the aminoacids forming coordination bonds with the metal ion (the metal ligands), a relatively large number of residues in the metal environment (≥20), and a distinct pattern of conservation of Cys and His residues in the site. Adventitious sites, on the other hand, tend to have a low number of donor atoms from the polypeptide chain (often one or two). These observations support the evaluation of the physiological relevance of novel metal-binding sites in protein structures.

## Introduction

More than one third of the entries in the Protein Data Bank contain at least one metal ion,^1,2^ while it has been estimated that no less than 40% of enzymes require metal ions for their biological function.^3,4^ Indeed, it is well known that a variety of metals are essential to life.^5,6^ The reactivity and physiological role of metal ions in metalloproteins is largely determined by the local protein structure environment through the modulation of how the metal is positioned in the active site, of how it interacts with the substrate and, for redox-active metals, of its reduction potential.^7,8^

About 88% of all structures in the Protein Data Bank (PDB) have been solved by X-ray crystallography.^9^ There are mainly two recurring issues that occur in the evaluation of metal-binding sites (MBSs) in these biomolecular structures: evaluating the chemical identity of the bound metal ion and ascertaining whether the observed site is physiologically relevant or is an artifact due to experimental conditions. Regarding the first point, it is known that sample preparation procedures, contamination by unintended metals, or experimental conditions, such as pH or irradiation, can affect the occupancy of MBSs.^10^ As an example, particle induced X-ray emission (PIXE) measurements on a sample set of 32 metalloproteins from structural genomics projects highlighted the presence of protein-bound metal ions that were not included in the deposited PDB structure.^11^ The ambiguity of the identity of the metal ion present in the MBS can also hamper the local quality of the 3D environment, leading to distorted geometries and other inaccuracies.^12−15^

Various extensive analyses of MBSs are available in the scientific literature,^16−22^ which focused on the properties of well-defined, biologically relevant sites. Instead, in this work, we will concentrate on the comparison of physiological vs adventitious MBSs, explicitly addressing the second issue mentioned in the previous paragraph. As a rule of thumb, previous literature suggested that adventitious sites tend to occur at the protein surface and have metal coordination numbers (CNs) on the lower side of the distribution of CNs for all sites of a given metal.^23,24^ To better circumstantiate these assertions, and possibly quantify them, we used the annotations of the zinc- and mononuclear iron-binding sites in the MetalPDB database^2^ to separate the physiological and adventitious ones, thereby creating a reference dataset. We leveraged this resource to train a classifier only on zinc-binding sites, using a deep learning (DL) approach.^25^ DL is becoming increasingly popular in structural bioinformatics, not only for the prediction of 3D protein structures^26,27^ but also to support the analysis of experimental structures.^28^ Two relevant recent examples are the identification of water interaction sites^29^ and, even more relevant to this work, the classification of enzymatic vs non-enzymatic metal ions in proteins.^30^ Our neural classifier was able to identify physiological sites for both zinc- and iron-binding sites, which are both transition metal ions, with very good accuracy. The analysis of the relative importance of the different features in driving the performance of the neural network suggested general properties of physiological MBSs.

In summary, the present work provides the community (i) with an extensive, organized dataset of annotated physiological/adventitious metal sites, which can be reused in other structural bioinformatics studies of metalloproteins, as well as (ii) with a freely available tool enabling non-experts to analyze new MBSs. Our analysis pinpointed some crucial properties defining the profile of physiological sites, which may be generally relevant at least for transition metal ions.

## Methods

### Preparation of the Dataset

MetalPDB^1^ contains information for all metalloproteins archived in the Protein Data Bank.^31^ For each metalloprotein, all its MBSs are automatically extracted according to the following procedure: for each metal atom in the structure, the non-hydrogen atoms at a distance smaller than 3.0 Å are identified as its donor atoms (shown in red in Supporting Figure S1), i.e., the atoms that bind directly to the metal. The protein residues or small molecules that contain at least one donor atom are the metal ligands (shown in cyan in Supporting Figure S1) and constitute the first coordination sphere of the metal ion. The full MBS (called minimal functional site in ref (2)) is obtained by including any other residue or chemical species having at least one atom within 5.0 Å from a metal ligand (shown in pink in Supporting Figure S1). MBSs describe the local structural environment around a metal ion or metal cofactor and do not depend on the overall macromolecular structure.

We started from the clusters of “equivalent sites” already available in MetalPDB.^1^ Two sites are equivalent if (i) they are found in PDB chains with the same structure (based on Pfam domain composition or on the sequence identity between the two chains being ≥50%) and, after structural superposition of the PDB chains, they (ii) are superimposed with the same metal atoms in the same positions. By construction, each cluster contains MBSs with a specific metal ion, i.e., metal-substituted sites are assigned to distinct clusters. This clustering procedure, which is similar to what is done in ref (30), allows redundancy to be removed from the dataset (with the exception of proteins having multiple MBS, as described below).

We made functional annotations available through the public MetalPDB interface for several of the above sites.^2^ We have been producing functional annotations of several of these sites through a manual protocol since shortly after the first release of the database in 2012.^1^ At the end of 2017, the annotations available through the public MetalPDB interface covered 17% of all zinc sites and 86% of all iron sites in MetalPDB.^2^ In the subsequent years, we have continued to extend the annotation of all metal sites through the same manual protocol in order to assemble the present dataset. All annotations are based on the analysis of relevant scientific literature, as follows: if the relevant article(s) describe the function of the metal (zinc or iron in this work), then the site is annotated as physiological; if no role is described for the metal but the experimental section reports that it was present in the purification or crystallization buffer, then the site is annotated as adventitious; in all other cases, the metal is annotated as “unknown role”, and that site was not used in this work. We further discarded all sites with no donor atoms from the protein. The large majority of the annotations for zinc and iron sites were motivated by and carried out in specific projects or collaborations.^32−35^

First, we randomly extracted one site for each cluster containing either zinc or individual iron ions (except heme sites). Then, for each selected site, we extracted the protein chain to which the site belongs and used it as an input to PROMOTIF^36^ and NACCESS^37^ to respectively calculate secondary structures and solvent accessibility (without taking into account the presence of the metal ion) at the residue level. A multiple sequence alignment (MSA) was generated for each protein using HHblits v. 3^38^ from the HH-suite^39^ to search the UniClust30 v. 2018-8 database^40^ with the parameters “-diff inf -id 99 -cov 50 -n 3”. This corresponds to the first stage of the DeepMSA protocol;^41^ we did not perform the additional stages of the procedure, suggested when the depth of the MSA is relatively low (for *N_f_* < 128), as initial tests on a subset of sites did now show any appreciable improvement in the final performance. For each residue of the protein chain, we included the following groups of features: (i) the profile of sequence conservation (fractional occurrence of each of the 20 amino acids), where the *i*-th row of the position-specific frequency matrix (PSFM) derived from the MSA was used as the representation of the *i*th position in the sequence; (ii) absolute and relative solvent accessibility; (iii) role in the MBS (2 for metal ligands; 1 for all other MBS residues; 0 for all other residues in the protein) (iv) secondary structure (helix/sheet/turn/other). This defined a set of 29 features for each residue in the protein (Supporting Table S1), leading to an input matrix of size *L* × 29 for each MBS, where *L* is the length of the protein chain harboring the site. The PSFM contains the frequency of each of the 20 amino acids at all the positions of the MSA where the majority of the sequences do not have gaps.

Note that proteins harboring more than one MBS will give rise to as many clusters as the number of MBSs. The definition of MBS in MetalPDB mandates that independent MBSs must have all distinct metal-binding protein residues. Therefore, even if a given chain may appear more than once in the dataset, the features describing the location (group i above) of its various MBSs and, consequently, the importance that the neural network gives to all the features along the sequence, will differ for the different sites.

The list of sites with their annotation is available as Supporting Information; the latter includes also the values of the features for all sites.

### Neural Classifier

**Figure 1:**
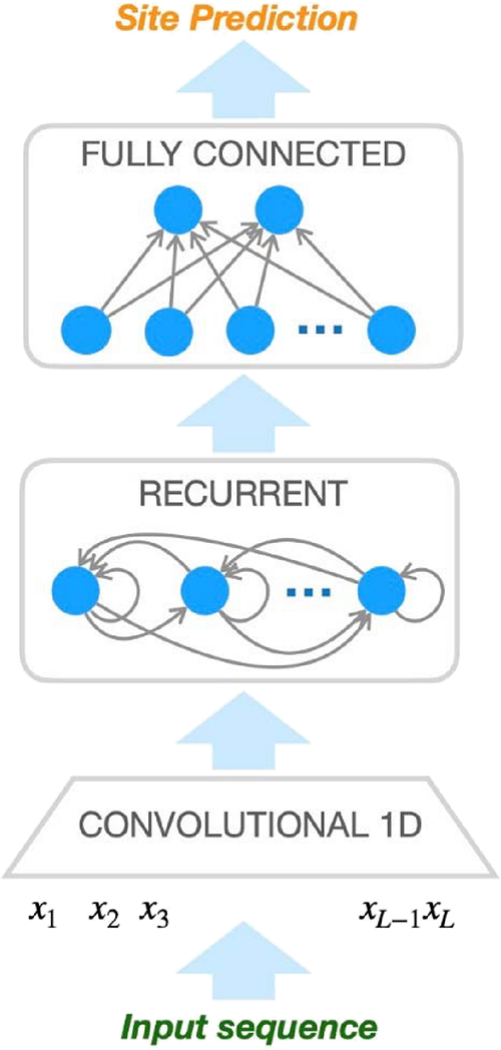
Scheme of the classifier. The network is composed by three modules. The convolutional module processes the input data, and its outcome is then fed to the recurrent module; finally, the fully connected module generates the estimated class probabilities for the input site.

Figure 1 shows the building blocks of the neural architecture and how they interact with each other. The input is a sequence *S* comprising *L* data points (*x*_t_). *L* is the length of the protein sequence, *x*_t_ represents the set of the *n* input features for the *t*-th residue, as described in the previous section. Thus, the input is an *n* × *L* matrix. After processing all the blocks, the classifier returns a prediction of the type of MBS that is contained in the protein structure.

Since we know that the role of the residues in the protein is strongly influenced by their neighborhood, in the first step, the classifier extracts information at a local level. The module Convolutional 1D generates a new representation of the sequence, *S*′, where the value of each position is a function of its neighborhood in *S*. This is done by performing a convolutional operation on *S* using a convolutional filter of size w (*k_w_*), corresponding to the size of the neighborhood that we take into account. Thus, *S*′ = {*x_t_*′; *t* = 1, ..., *L*} where *x_t_*′ = *f*_CONV_ (*x_t_*, *k_w_*). As we are in a machine learning context, this module learns to extract local information from the sequence, which is done by learning the convolutional function *f*_CONV_ and, more specifically, the values of the convolutional filter *k_w_*.

A suitable network model to deal with sequential data, such as *S*′, is the recurrent neural network (RNN). In RNNs, the model creates a representation *h_t_* for the *t*-th residue of the chain, which depends on the representation generated for the preceding residue, so *h_t_* = *f*_RNN_ (*x_t_*, *h*_(*t*-1)_). This implies that the representation of each residue depends on all the preceding ones. This property of the RNN makes it a suitable tool for extracting a global representation of the input sequence. The RNN is fed with the output of the convolutional 1D block, *S*′, and generates a sequence of residue representations *h*_1_, *h*_2_, ..., *h_L_*. In practice, we are only interested in the last representation, *h_L_*, which constitutes the global representation of the whole sequence and, thus, is the outcome of the RNN module.

At this point, we have an array *h_L_* representing the input sequence *S*, whose size does not depend on the length *L* any longer. *h_L_* is the input to the final layer of the classifier (fully connected), whose aim is to associate one of the two classes to the input sequence *S* (now represented by *h_L_*). This is achieved by approximating a probability distribution over the classes, given the input, as the outcome of the layer, *y* = [*P*(physiological*|S*), *P*(adventitious*|S*)]. The resulting predicted class is the one having the highest probability value as computed by a linear feedforward layer with two output neurons.

All the layers described above are neural blocks, whose functions are learned as in the classical learning process using the backpropagation algorithm.^42^ A complete description of the network architecture, all related parameters, hyperparameters, and training strategy is available in the Supporting Information.

### Evaluation of Feature Importance

*S_i_* = (*x*_*i*,1_, *x*_*i*,2_, ..., *x*_*i*, *L*_) is the *i*-th row of *S*, i.e., the ensemble of the values of the *i*th feature for all the residues in the sequence. We define a perturbed version of *S_i_* as *S*_i_^p^ = *S_i_* + α*_i_g*, where α_*i*_ is the magnitude of the domain of the *i*th feature and  is an array of Gaussian noise *N*(0,1) samples. We then define: *Y*_targ_ = {*y*_1_, ..., *y_N_*}, *Y* = {*y*_1_, *y*_2_, ..., *y_N_*}, *Y*_noise(*i*)_ = {*y*_1, noise(*i*)_, *y*_2, noise(*i*)_, ..., *y*_*N*, noise(*i*)_} where *Y*_targ_ contains the target values, *Y* contains the predictions with the original input dataset (*S_i_*), and *Y*_noise(*i*)_ contains the predictions generated after the perturbation of the *i*th feature for all residues (*S_i_^p^*). If Acc()∈[0,1] is the accuracy of the prediction, we define the importance of the *i*th feature as *I_i_* = Acc(*Y*_targ_, *Y*) – *Acc*(*Y*_targ_, *Y*_noise(*i*)_). The more sensitive the model is to variations of the *i*th feature, the greater is the impact on the accuracy of the predictions and, hence, the value of *I_i_*. These statistics were calculated on the test sets of the 10-fold cross-validation procedure (Supporting Information).

## Results

### Zinc Dataset and Features

We annotated one representative site for each zinc–protein family in MetalPDB.^2^ These sites were taken from structures solved using any technique; for X-ray and cryoEM structures, we did not use a resolution filter. Our dataset of zinc-binding sites comprised 1944 physiological sites and 3352 adventitious sites.

Our work started from the hypothesis that it is possible to pinpoint a restricted number of key structural properties that, together with sequence information, determine the nature (physiological or adventitious) of the metal-binding site (MBS hereafter). We thus aimed at building a classifier to predict the type of MBS, and, in line with our hypothesis, understand/discover whether a sequential representation of a well-defined set of features was sufficient to successfully accomplish this task. We adopted a combination of features focused on the structural properties and features encompassing the entire protein sequence. The protein structure provides information on which protein residues constitute the first coordination sphere of the metal ion and its local environment (defined in the binding role features) and on the secondary structure and solvent accessibility of all protein residues, whereas multiple sequence alignments (MSA) quantify the conservation of the protein chain harboring the site. The MBS is a relatively small portion of the entire 3D structure, and its associated features can act as a weight for the different parts of the sequence from the binding role perspective, even though we did not explicitly instruct the classifier to do so.

To train the neural network, we used all zinc(II) sites in the dataset; the contribution of the physiological sites to the cost function of the classifier was scaled up by a 1.7 factor to account for the imbalance with respect to adventitious sites. The training involved a *k*-fold cross-validation^43^ procedure (with *k* = 10) for which the dataset was divided in 10 subdatasets, commonly referred to as folds. Each fold is expected to be representative, from a data distribution perspective, of the whole dataset. Out of these 10 subdatasets, one is kept as the holdout dataset and used as the test set, one is used as the validation set, and the remaining eight folds are used to optimize the network parameters. In practice, the parameters are adjusted to optimize the classification of the validation set. In our configuration, the average sequence similarity between each fold and all other folds was 22.5% ± 0.1% (which thus corresponds to the average similarity between any given test set and the corresponding training set). After optimization, the performance of the model for a given fold selection is computed based on the classification of the test set, which has not been used until this point of the procedure and, thus, is completely new to the classifier. The procedure is repeated *k* times by rotating the role of all folds (training, validation, and test).

### Performance of the Zinc(II) Neural Classifier

We obtained an average accuracy (fraction of all sites that were correctly identified over the 10 test sets) of 89.9% ± 1.3%. The sequence similarity of the test sets with respect to the corresponding training sets was sufficiently low to ensure that the observed performance was not biased by the occurrence of close homologues in the different groups. To understand how the model behaves on the two classes (physiological or adventitious in this case), we computed the confusion matrix (Table 1) from which a number of performance metrics can be derived (Table 2). The MCC value, a measure of overall performance that is not particularly sensitive to the different sizes of the positive and negative datasets,^44^ of 0.780 indicates that the neural classifier has a balanced performance. More in detail, our classifier appears to be slightly better at identifying adventitious than physiological sites. The predictions of physiological sites had an 11.4% error (false discovery rate, FDR). This error rate is somewhat higher than the misannotation rate, which we estimate to be about 5% by double checking a random selection of 100 sites.

**Table 1:** Confusion Matrix of the Performances for the Test Sets Averaged over the 10-Fold Cross Validation Procedure^a^

	estimated physiological	estimated adventitious
real physiological	1615 TP	329 FN
real adventitious	208 FP	3144 TN

a Each row corresponds to the data points belonging to a certain class (“real” class, corresponding to physiological/adventitious zinc(II) sites in this work), whereas the columns show how the model classified the points (“estimated” class).

**Table 2:** Performance Metrics Derived from the Results of Table 1

metric	value	formula	meaning
PPV	0.886	TP/(TP + FP)	fraction of positive predictions that are correct
recall, TPR	0.831	TP/(TP + FN)	fraction of all positive sites that are correctly classified
NPV	0.905	TN/(TN + FN)	fraction of negative predictions that are correct
specificity, TNR	0.938	TN/(TN + FP)	fraction of all negative sites that are correctly classified
FDR	0.114	1-PPV	fraction of positive predictions that are wrong
MCC	0.780		Matthews’ correlation coefficient

Binary classifiers assign each data point to a class based on their computed score with respect to a threshold β (typically β = 0.5 for classifiers using the 0–1 score range as done here). Thus, for different values of β, we have different associated confusion matrices. The ROC curve is the set of pairs [1-TNR(β), TPR(β)] obtained by varying the threshold β.^45^ In practice, the ROC curve plots TPR as a function of 1-TNR. The ideal classifier should have 1-TNR = 0 and TPR = 1 (corresponding to the top-left corner of the plot). The area under the ROC curve (AUC) quantifies the performance of the tool; the larger the AUC, which ranges from 0 to 1, the better. The average ROC curve over the 10-fold cross validation procedure (Supporting Figure S2) shows that the behavior of our classifier was very similar for all the folds and clearly distant from a random classifier. The AUC was 0.940 ± 0.006.

**Figure 2:**
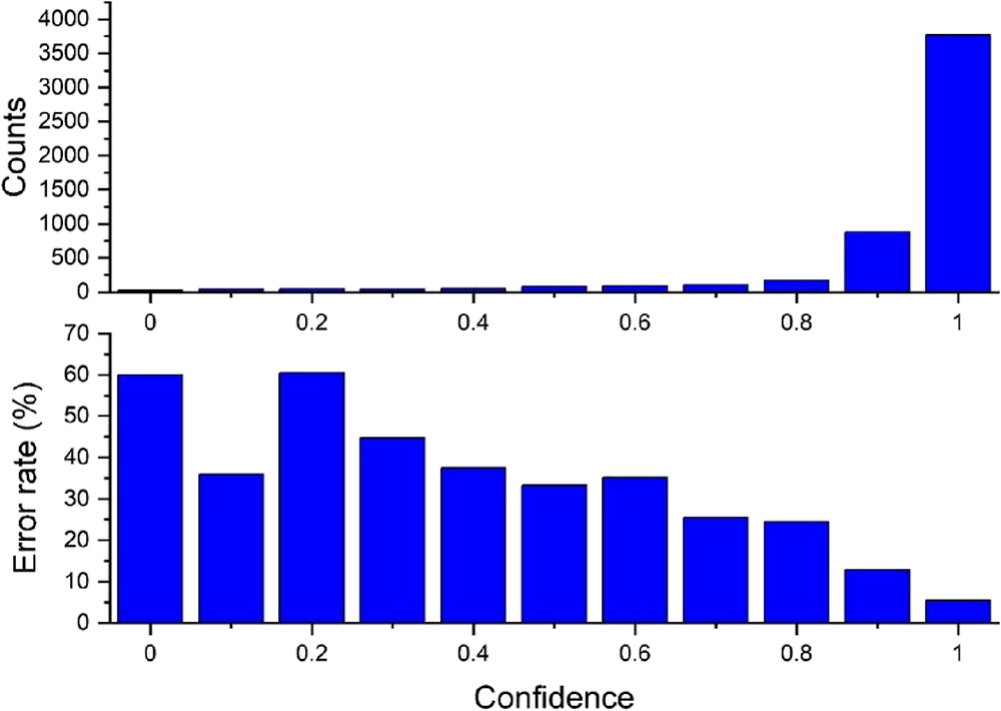
(Top) Number of predictions for zinc(II) sites with a given confidence (absolute value of the difference between the score of the positive and of the negative classes). (Bottom) Error rate of the neural classifier in each confidence range. The data have been computed using 0.1 bins.

For any given site, the score assigned by the neural classifier to each of the two output classes can be regarded as the estimated probability that the site belongs to either class. The absolute value of the difference between the two scores is thus a measure of the imbalance with which the classifier predicts a site to be physiological or adventitious. We refer to this difference as the “confidence” of the prediction. As shown in Figure 2, the large majority of zinc(II) sites (88%) are classified with a confidence higher than 0.85. The error rate of the neural classifier is also strongly dependent on the confidence of the prediction. Indeed, low-confidence predictions have error rates between 30 and 60%, whereas the error rate for predictions having a confidence between 0.85 and 0.95 is 13% and the rate when the confidence is higher than 0.95 is as low as 5.4%. By grouping together all 4652 predictions with a confidence higher than 0.85, we have an error rate of 6.9%.

In our protocol, we used the information from the asymmetric unit of the crystal structure without attempting a reconstruction of the structure based on symmetry information. Furthermore, for MBSs at a protein–protein interface, we computed the features using only a single chain even if both chains were present in the asymmetric unit. This choice was due to the difficulty of properly building deep multiple sequence alignments for pairs of interacting proteins. Nevertheless, the performance of the neural classifier for interfacial sites was marginally lower than for all other sites.

### Evaluation of the Results

**Figure 3:**
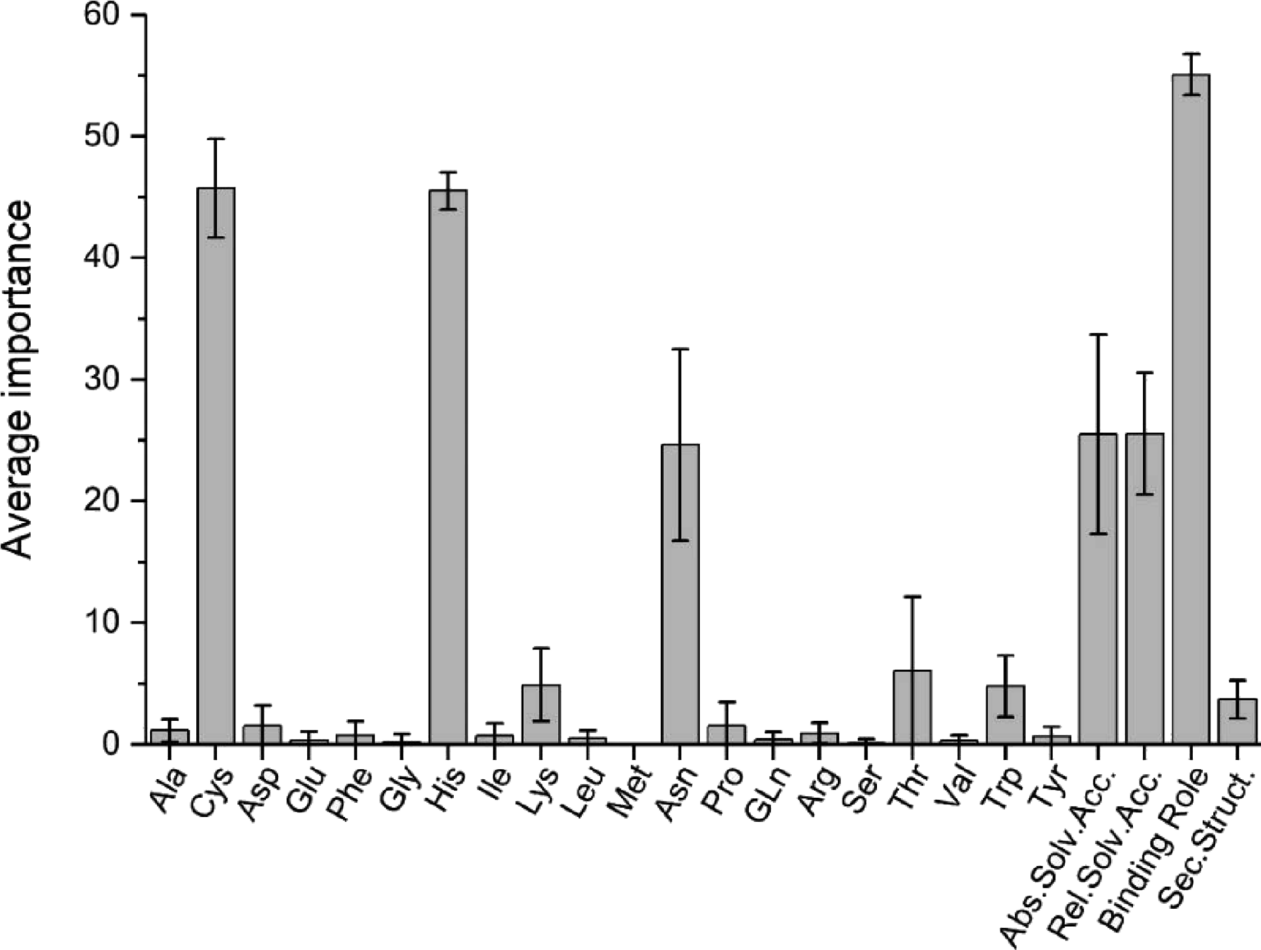
Importance of the input features. The plot shows the decrease in classification accuracy caused by the perturbation of the input features of the test sets, measured by the importance parameter (see Methods) averaged over the ten folds. The 20 amino acids were perturbed individually. Features describing the binding role of the residues and their secondary structure were merged.

Since our classifier is a neural network, it is not possible to rationalize its decisions.^46,47^ Nevertheless, we tried to obtain some insight by perturbing the input features in order to evaluate the corresponding impact on the predictions, thus revealing the importance of each of them from the network perspective. We did this by adding Gaussian noise to one feature at the time in the test sets (thus degrading the quality of the input to the network) and observing the decrease of classification accuracy (Figure 3). The most important feature is the identification of which amino acids belong to the first and second coordination spheres of the metal (binding role). This is followed closely by information on the conservation profiles of Cys and His, essentially at the same level. Other significant impacts are those of solvent accessibility, either absolute, Abs.Solv.Acc., or relative, Rel.Solv.Acc.^37^ The conservation profile of Asn has some importance. Notably, information on secondary structure elements has a negligible impact on the performance of the classifier (Figure 3).

We further analyzed the above results by training the same neural classifier with a reduced set of input features. For this experiment, we used the following groups: (i) the sequence conservation of Cys, His, Asn, and Thr (the four most relevant residues in Figure 3); (ii) the three binding role features; (iii) the two solvent accessibility features; and (iv) various combinations of (i), (ii) and (iii). The results confirmed the prominent relevance of the binding role information, closely followed by the conservation of four selected amino acids (Table 3). This analysis deemphasized the importance of solvent accessibility. However, it should be noted that we did not perform a search for the best NN architecture for each combination of input features, making the results of Table 3 a lower limit for the performance of the corresponding optimal network architecture.

**Table 3:** Average Accuracy over the Test Sets of the 10 Fold Cross-Validation Procedure for Neural Networks Trained with a Subset of the 29 Features

features	test accuracy	standard deviation
conservation of Cys, His, Asn, and Thr	80.9	1.5
binding role	84.5	1.5
solvent accessibility	64.0	4.9
conservation of C, H, N, T plus binding role	86.6	1.6
conservation of C, H, N, T plus solvent accessibility	75.7	3.3
binding role plus solvent accessibility	84.2	1.9

**Figure 4:**
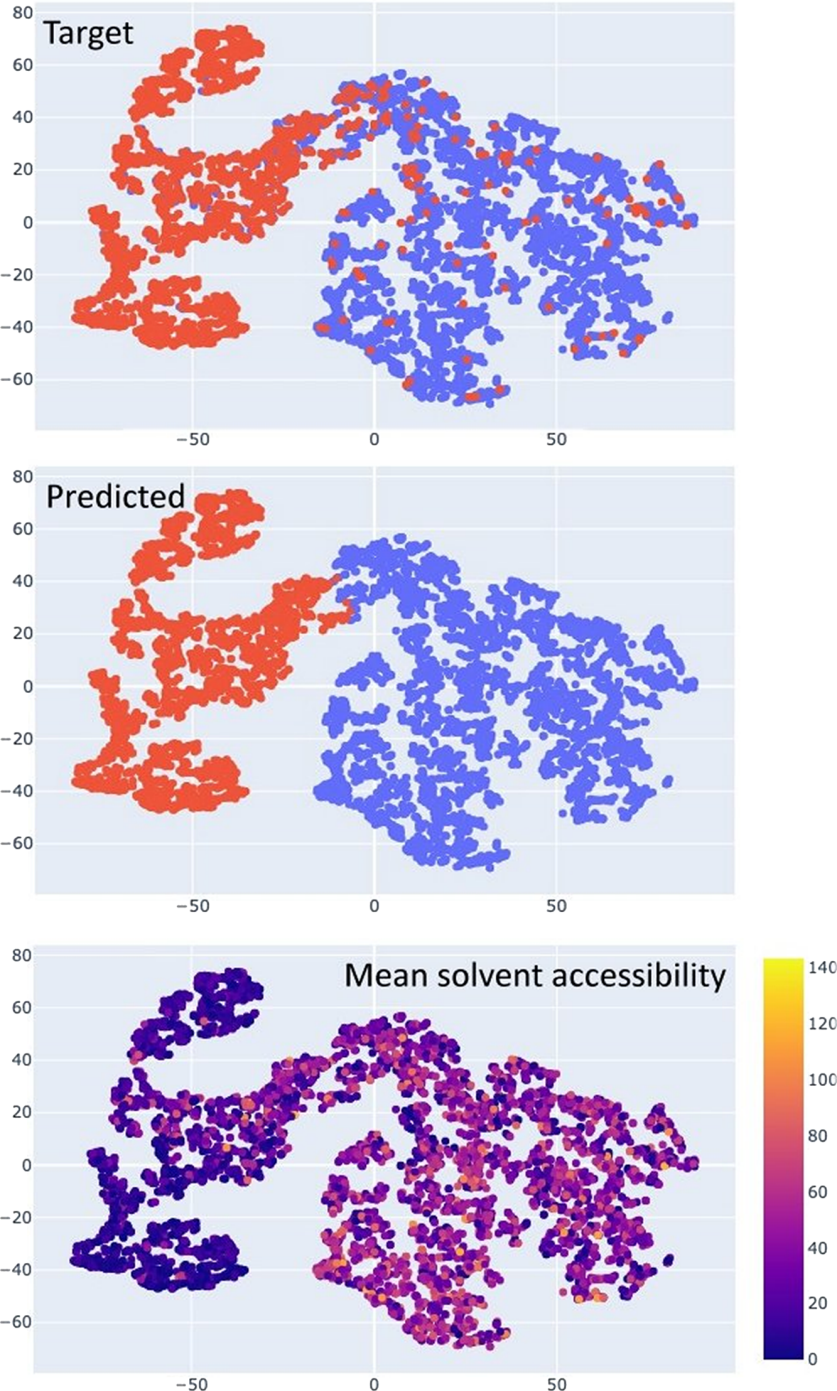
Data visualization generated with the TSNE dimensionality reduction algorithm. This algorithm produces a representation in an arbitrary 2D space of the distance between the points in the original multidimensional space of the data representation of the neural network. The points (red: physiological sites; blue: adventitious sites) are colored according to the (top) known class and (center) predicted class. In the bottom panel, the points are colored based on the average absolute solvent accessibility of the protein residues providing the donor atoms to the zinc(II) ion(s) regardless of their classification.

Figure 4 shows a visualization of the input data in the form of a reduced-dimensionality plot of the data representation learned by the network (i.e., the *h_L_* vector generated by the RNN module), colored based on the input class (Figure 4A) and predicted class (Figure 4B). The neural classifier achieved a clear separation between the predicted physiological and adventitious sites. Thus, from the machine perspective, the large majority of the points belonging to a given class are closer to each other than to the points of the other class. This separation is a very good match to the distribution of the real data (Figure 4A). This is the result of a complex interplay among all the features analyzed in the previous sections. Nevertheless, it is possible to relate the data representation learned by the network to the experimentally determined properties of the MBSs, also based on the information on feature importance. For example, by highlighting the accessibility of the sites, we observed that low-accessibility sites lie in the region that comprises the majority of the physiological sites (Figure 4C).

### Application of the Zinc(II) Neural Classifier to Non-Heme Mononuclear Iron Sites

We applied the zinc(II) neural classifier to 451 non-heme mononuclear iron sites taken from MetalPDB in order to investigate whether the differences between the coordination chemistry of zinc and iron would significantly affect the performance. We did not take into account heme proteins because these cofactors have highly specific characteristics. Iron(II) and iron(III) sites were used without distinction. The results were good, with 78.6% of the physiological sites and 78.3% of the adventitious sites correctly identified (Table 4). The error rate for positive predictions was 10.8%, which is practically the same as for the zinc sites. Seventy-three percent of the predictions had a confidence value of 0.85 or more.

**Table 4:** Confusion Matrix for the Classification of Non-Heme Mononuclear Iron Sites by the Zinc(II) Neural Classifier

	estimated P	estimated N
real P	246 TP	67 FN
real N	30 FP	108 TN

### Experimental Determinants of the Performance of the Neural Classifier

**Figure 5:**
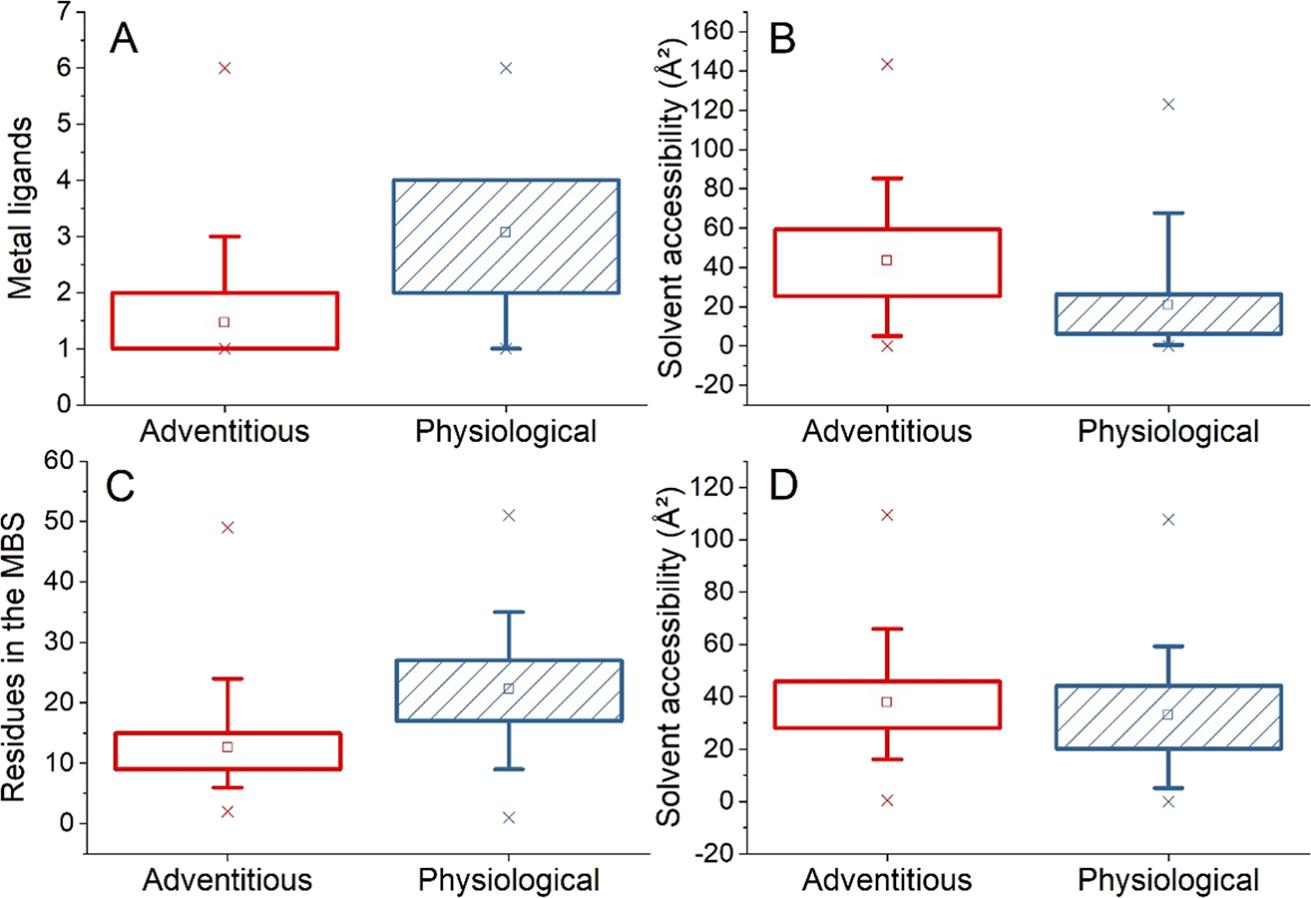
Comparison of value ranges (adventitious vs physiological) for a selection of the features defined for all zinc-binding sites. (A) Number of amino acids binding the metal (“metal ligands”). (B) Average absolute solvent accessibility of the metal ligands. (C) Number of residues in the site. (D) Average absolute solvent accessibility of the residues in the second coordination sphere. Red empty boxes: adventitious sites; blue hatched boxes: physiological sites. Box plot setup: the box goes from the 25th to the 75th percentile (1st and 3rd quartile, respectively); whiskers are at the 5th and 95th percentile; the minimum and maximum values are shown by crosses; the square in the box corresponds to the mean value.

Figure 5 shows the distribution of the features highlighted in Figure 3 for the residues of the zinc(II) MBSs in the whole dataset. First, we inspected the binding role of residues in the MBS, which was the most important group of features. It can be immediately observed that physiological and adventitious sites differ in the distributions of the number of metal-binding amino acids (“metal ligands”), as well as for their average solvent accessibility (Figure 5A,B). Adventitious sites tend to have a lower number of metal ligands, which are more solvent-exposed than in the case of physiological sites. However, the distribution of solvent accessibility values for the latter sites is relatively wide. When looking at the whole MBS, physiological sites involve a larger number of residues than adventitious sites. Per construction of the MetalPDB database, the MBS is the ensemble of the atoms in the metal ligands and any other atom belonging to a chemical species within 5 Å from a ligand;^1^ the MBS describes the local 3D environment around the metal cofactor independently of the protein fold. The average number of residues in the MBS is 22.3 vs 12.5 for physiological and adventitious sites, respectively (Figure 5C). Notably, the amino acids in the second coordination sphere tend to have the same solvent exposure in both groups of sites (Figure 5D), with values similar to those of the metal ligands in adventitious sites. As a result, the average solvent accessibility of the whole MBS is alike for both types of sites (not shown).

## Discussion

We trained a convolutional-recurrent deep neural network^48−50^ to classify physiological and adventitious zinc(II)-binding sites (Supporting Figure S3) in proteins using a consolidated machine learning approach.^25^ Adventitious sites are the result of the experimental conditions under which the protein sample was prepared and crystallized and are expected not to be populated in vivo. We constructed the dataset for training and testing the neural classifier using the manually annotated entries in the MetalPDB database.^2^ Sequence and structure comparison methods were used to remove redundancy from the dataset. Our zinc(II) neural classifier achieved a satisfactory performance, quantified by a MCC value of 0.780, which is in line with the performance of other DL applications in structural biology,^29,30^ and a AUC of 0.94. Only about 11% of the sites classified as physiological were incorrectly assigned, whereas the error rate for the classification of adventitious sites was slightly better (9.6%). The network outputs two independently estimated scores for each site, practically corresponding to the probability that the site is adventitious and the probability that the site is physiological. The absolute value of the difference between the two scores can be taken as a measure of the confidence that the classifier has in its assignment of a specific site to either class. In fact, the error rate for predictions with a confidence of 0.85 or greater is somewhat smaller than for lower confidence values (7% vs 30–50%). A lower confidence for a given data point reflects the fact that the point is located close to the surface separating physiological and adventitious sites (Figure 4), which indeed corresponds to a lower reliability of the prediction. The neural classifier featured a satisfactory performance also for non-heme mononuclear iron sites (78% accuracy), which had not been used at any stage of its development. This is remarkable given the negligible protein sequence similarity between the two groups and the different coordination preferences of zinc(II) vs iron. For example, in our datasets, the majority of zinc(II) sites have a coordination of 4, whereas the most common coordination number for the analyzed iron sites is 6 (e.g. Supporting Figure S3D). The results on iron sites constitute both a further validation of the implemented approach and an intriguing outcome of our work.

The satisfactory performance of the neural classifier allows it to be used as a tool to validate novel metal sites binding zinc(II) or mononuclear iron ions, which are both transition metals. Beyond this achievement, by looking at how the different features affected the classification, we obtained insight on the chemical properties of physiological and adventitious sites. This process was eased by the relatively straightforward definition of the features we input to the network. Figure 3 shows that pinning down the metal ligands as well as the protein residues in the MBS constitutes the most important input to achieve a high-quality classification. Using only these features to train the network results in an accuracy of 84.5% (Table 3). From the structural viewpoint, the next most important property is the solvent accessibility of the metal ligands. At the sequence level, the conservation pattern of Cys and His residues are crucial. Information about conservation of Cys and His, along with Asp and Glu, has been extensively used for the sequence-based prediction of the occurrence of metal-binding sites.^51−54^ Intriguingly, information on Asp and Glu did not have a significant impact on the performance of the neural classifier, whereas the conservation of Asn played some role. The latter finding is difficult to rationalize: the only indication we have is that Asn is about 1.6 times more common in the second sphere of physiological sites than adventitious sites. Based on the above hints, we analyzed in detail the experimental dataset and were able to define some specific trends that can constitute rules for the identification of physiological sites. These trends support the anecdotal evidence in the scientific literature that adventitious sites occur at the protein surface.^23,24,55^ A likely reason for this is the fact that the protein is fully folded before the metal ion is captured e.g., from the crystallization buffer.

In detail, the protein provides less metal ligands in adventitious sites than in physiological sites; in addition, the ligands in the former sites show higher solvent accessibility in agreement with the surface location of adventitious sites (Figure 5). Consequently, the first coordination sphere of adventitious sites is more likely to involve water molecules or other non-proteinaceous ligands and has higher local B-factors, potentially hampering the detection of all metal ligands.^23^ Another noteworthy difference among physiological and adventitious sites, which is also dependent on their different location within the protein structure, is in the number of residues in the site. According to the MetalPDB protocol,^1^ the MBS comprises the metal ligands together with any protein residue having at least one heavy atom within 5 Å from any atom of the ligands. Indeed, using only the information on which residues are in the MBS (binding role) allows the classifier to achieve an accuracy of nearly 85% (Table 3). As they are located deeper within the structure, physiological MBSs involve, on average, nearly twice the number of residues than adventitious MBSs.

To evaluate a possible usage scenario for our classifier, we selected 14 different zinc sites from protein structures released in 2022. For 11 of these (five physiological and six adventitious sites), we obtained predictions with a confidence greater than 0.50, which were all correct (Supporting Table S2). Whereas all the physiological sites had some sequence similarity to structures already contained in the dataset, the adventitious sites were all novel. An interesting example is that of a zinc(II) ion found in the active site of 1,2-β-mannobiose phosphorylase from *Thermoanaerobacter* sp. X-514 (PDB entry7FIS), bound to two protein residues and to the phosphate moiety of a molecule of the substrate mannose-1-phosphate.^56^ Enzyme activity assays show that the zinc ion is a result of crystallization conditions and is not required for catalysis. In agreement with this, the neural classifier predicted this MBS to be adventitious.

## Conclusions

We trained a deep neural network to classify zinc(II)-binding sites in the 3D structures of proteins as physiological or adventitious. In addition to achieving a very good performance for such sites, the classifier also had a remarkable accuracy for non-heme mononuclear iron sites. Using the hints provided by the analysis of feature importance, we managed to pinpoint some simple structural features that can be used as rules to distinguish physiological and adventitious sites. MBSs involving 20 protein residues or more (as computed by MetalPDB, Supporting Figure S1) are extremely likely to be physiological as well as sites with four metal ligands or more provided by the protein chain. In evaluating this aspect, one needs to be cautious of the inclusion of additional amino acids provided by sequence tags inserted, e.g., to facilitate protein purification (such as poly-His tags), which of course are not physiologically relevant. Another important parameter is the solvent accessibility of the metal ligands, although it is not practical in this case to define a reliable cut-off value because of the width of the corresponding distributions. Nevertheless, metal ligands in physiological MBSs tend to have low solvent accessibility. The above rules should apply at least to “simple” (mononuclear) sites harboring transition metal ions. Notably, the classification of complex metal cofactors, such as polymetallic clusters or organometallic cofactors, should be more straightforward than what we accomplished here, mostly because these sites are very unlikely to assemble in the absence of specific biosynthetic systems or of finely tuned chemical conditions during sample preparation.

The present classifier is freely available to the scientific community as a stand-alone tool (see Data and Software Availability) to enable the annotation of zinc and iron sites in the newly determined 3D structures of metalloproteins. This allows researchers to keep pace with the ever-increasing throughput of structural biology projects and makes functional analysis of metal sites possible even to non-experts in bioinorganic chemistry.

## Supporting Information Available

(Supporting Figure S1) Construction of MBSs; (Supporting Figure S2) ROC curve for the DL classification of zinc sites; (Supporting Figure S3) examples of successful predictions by the neural classifier; (Supporting Table S1) list of the 29 features representing each residue of the input sequence; (Supplementary Table S2) classification of a set of zinc sites extracted from PDB structures released in 2022; preparation and training of the neural network (PDF)
Table listing all physiological zinc sites used in this work (XLSX)
Table listing all adventitious zinc sites used in this work (XLSX)
Table listing all physiological iron sites used in this work (XLSX)
Table listing all adventitious iron sites used in this work (XLSX)
Pickle files for physiological and adventitious sites (ZIP)

## Notes

All data are available as Supporting Information. The code for the classifier is available from “GitHub - cerm-cirmmp/MBSDL: Metal Binding Sites Deep Learning”.
