# Supplementary material for: Learning to Identify Physiological and Adventitious Metal-Binding Sites in the Three-Dimensional Structures of Proteins by Following the Hints of a Deep Neural Network
Source: J Chem Inf Model. 2022 Jun 9;62(12):2951–60. doi: 10.1021/acs.jcim.2c00522 (PMC9241070; doi:10.1021/acs.jcim.2c00522)
Supplement: Supplementary file 1 — ci2c00522_si_001.pdf [file ci2c00522_si_001.pdf]

Supporting Information

Learning to identify physiological and adventitious metal-binding sites in the three-dimensional structures of proteins by following the hints of a deep neural network

Vincenzo Laveglia[1], Andrea Giachetti[1], Davide Sala[1,2,3], Claudia Andreini[1,3,4,*], Antonio Rosato[1,3,4,*]

1 Consorzio Interuniversitario di Risonanze Magnetiche di Metallo Proteine, Via Luigi Sacconi 6, 50019 Sesto Fiorentino, Italy.

2 Institute for Drug Discovery, Leipzig University, Brüderstr. 34, 04103 Leipzig, Germany

3 Magnetic Resonance Center (CERM), University of Florence, Via Luigi Sacconi 6, 50019 Sesto Fiorentino, Italy.

4 Department of Chemistry, University of Florence, Via della Lastruccia 3, 50019 Sesto Fiorentino, Italy.

Corresponding author
Antonio Rosato, Ph.D
Via Luigi Sacconi 6
50019, Sesto Fiorentino
Italy
rosato@cerm.unifi.it

Figures

Supporting Figure S1. Construction of MBSs. For each metal atom in any selected 3D structure, the non-hydrogen atoms at a distance smaller than 3.0 Å from the metal ion (blue sphere) are identified as its donor atoms (red atoms), i.e. the atoms that bind directly to the metal. The protein residues or small molecules that contain at least one donor atom are the metal ligands (cyan sticks), and constitute the first coordination sphere of the metal ion. The full MBS is obtained by including any other residue or chemical species having at least one atom within 5.0 Å from a metal ligand (pink sticks).

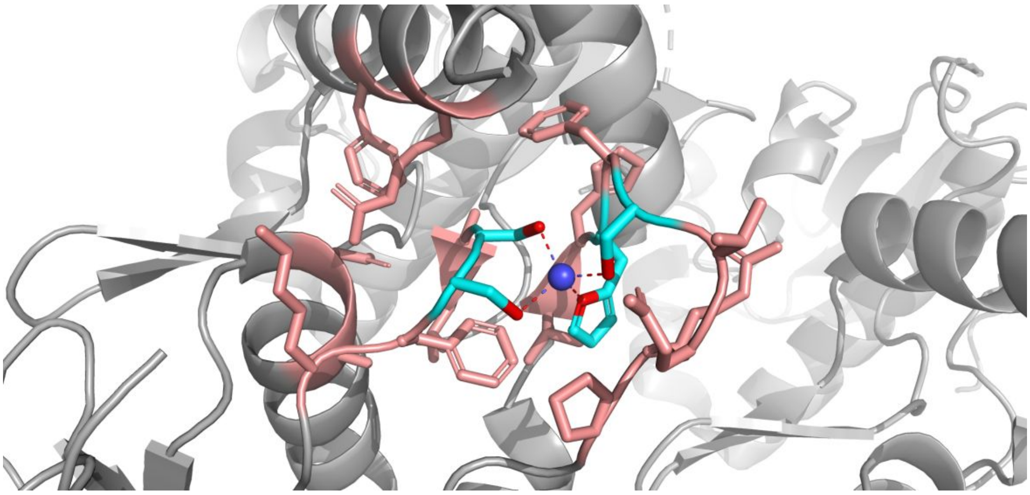

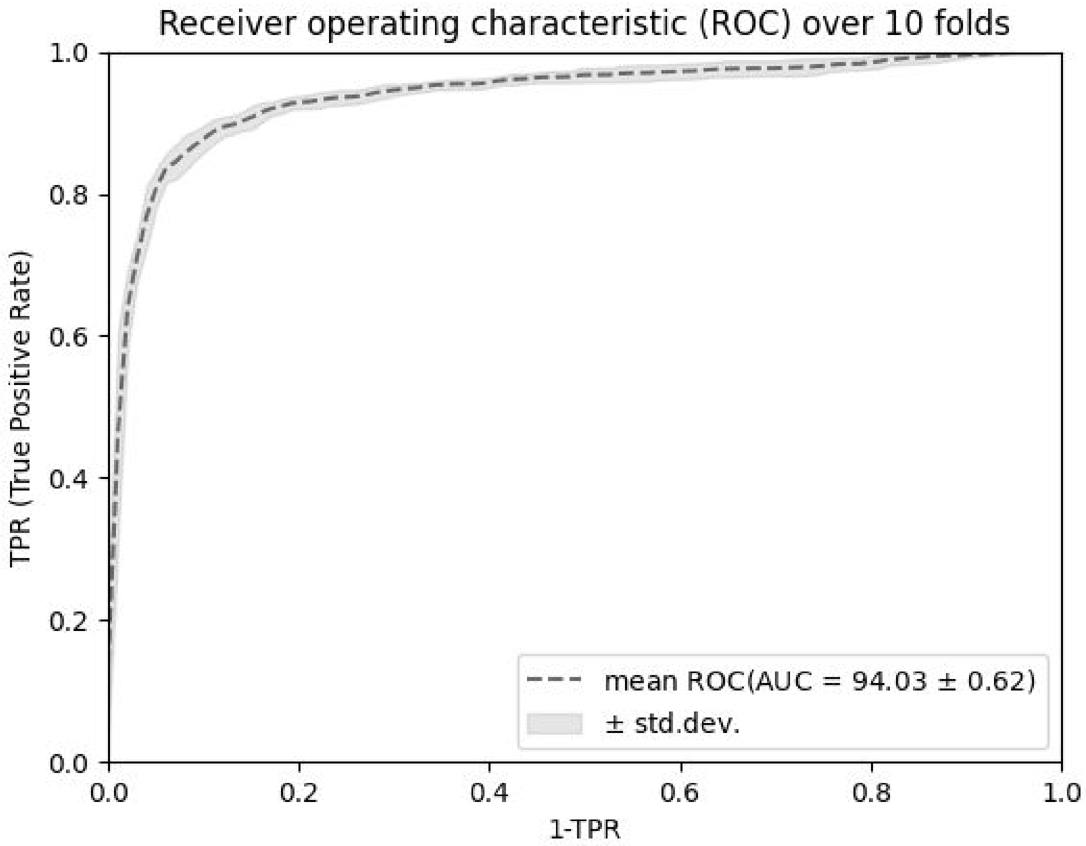

Supplementary Figure S2. ROC curve for the DL classification of zinc sites, averaged over the 10 test sets.

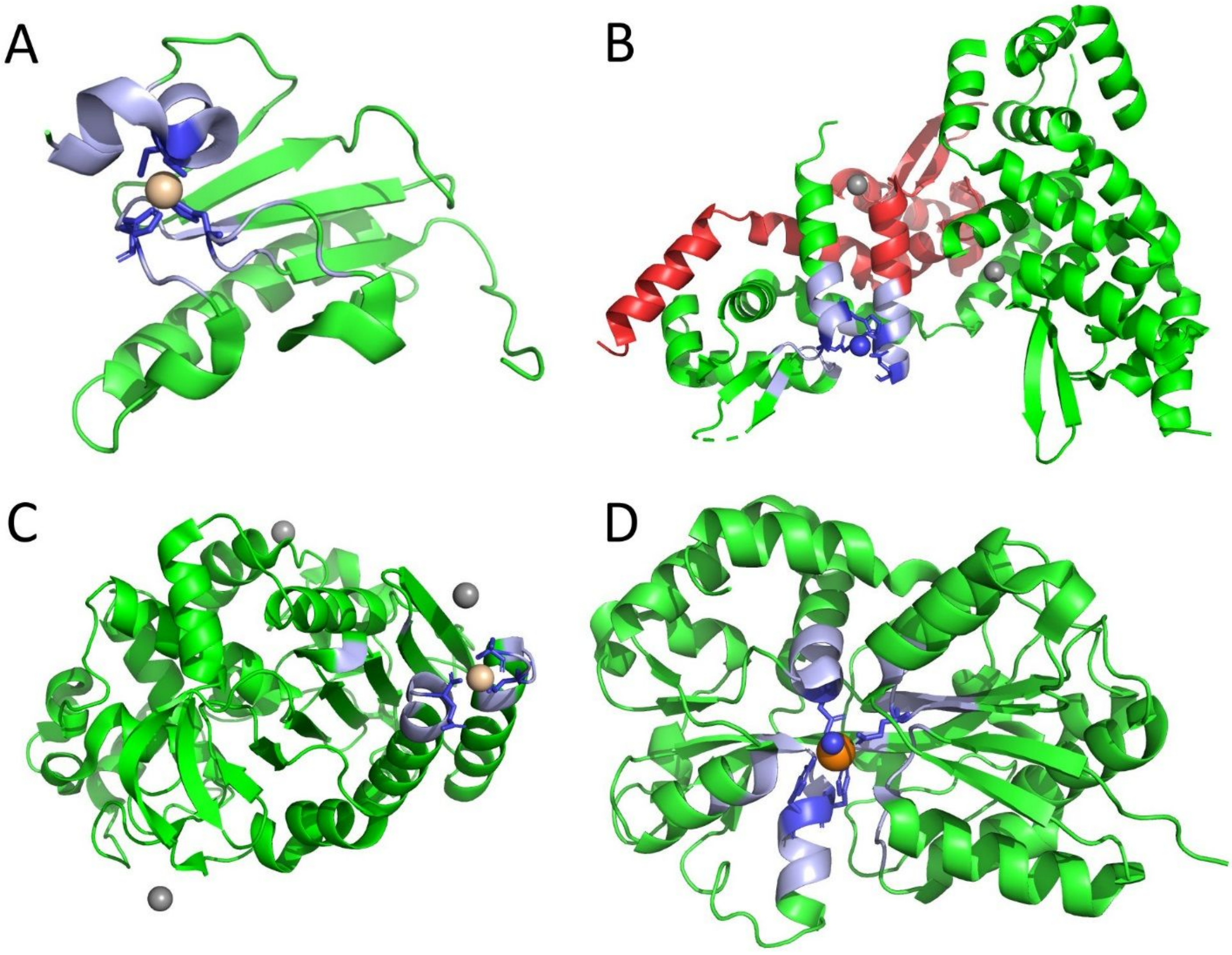

Supplementary Figure S3. Examples of successful predictions by the neural classifier. A) the physiological zinc(II) site of structure 3OXK[1]; B) one of the two physiological interfacial zinc(II) sites of structure 1U2W[2]; C) one of the adventitious zinc(II) sites of structure 5CHU[3]; D) the iron(III) site of structure 1VXV[4]. In all panels, the metal ion of interest is shown as a wheat (zinc) or orange (iron) sphere; the metal ligands are shown as blue sticks; the backbone of the MBS is colored in light blue. The protein backbone is displayed using a cartoon representation. In panels B-C other zinc(II) ions present in the structure are shown as grey spheres. In panel D, the small blue sphere is a water molecule coordinating the iron ion. In panel B, the two protein chains are colored in green and red respectively (except for the MBS).

Tables

Supporting Table S1. List of the 29 features representing each residue of the input sequence. The binding role features define the function of the residue within the MBS (2 for metal ligands; 1 for all other MBS residues; 0 for all other residues in the protein)

MSA score of Ala
MSA score of Cys
MSA score of Asp
MSA score of Glu
MSA score of Phe
MSA score of Gly
MSA score of His
MSA score of Ile
MSA score of Lys
MSA score of Leu
MSA score of Met
MSA score of Asn
MSA score of Pro
MSA score of Gln
MSA score of Arg
MSA score of Ser
MSA score of Thr
MSA score of Val
MSA score of Trp
MSA score of Tyr
Absolute solvent accessibility
Relative solvent accessibility
Binding role 0 (true/false)
Binding role 1 (true/false)
Binding role 2 (true/false)
Secondary structure: helix (true/false)
Secondary structure: sheet (true/false)
Secondary structure: turn (true/false)
Secondary structure: other (true/false)

Supplementary Table S2. Classification of a set of zinc sites extracted from PDB structures released in 2022. P() approximates the real probability distribution; because of the approximation, P(Adventitious)+P(Physiological) may differ slightly from 1.000.

**Site**	**P(Adventitious)**	**P(Physiological)**	**Confidence**	**Literature evidence**
7puj_A_7puj_1	**1.00**	0.00	1.00	Adventitious
7puj_A_7puj_2	**0.994**	0.00	0.994	Adventitious
7puj_A_7puj_3	**0.995**	0.002	0.993	Adventitious
7pso_C_7pso_1	0.003	**0.990**	0.987	Physiological
7fis_B_7fis_4	**0.990**	0.007	0.983	Adventitious
7puj_A_7puj_4	**0.960**	0.037	0.923	Adventitious
7q4o_G_7q4o_2	0.062	**0.928**	0.866	Physiological
7q4o_G_7q4o_5	0.085	**0.907**	0.822	Physiological
7qgj_A_7qgj_2	0.095	**0.900**	0.805	Physiological
7q4o_G_7q4o_3	0.177	**0.816**	0.639	Physiological
7fis_D_7fis_1	**0.764**	0.235	0.529	Adventitious

Additional methodological details: preparation and training of the neural network

Network Architecture, Hyper-parameters and Training

This section describes the architectural details of the neural network implemented in this work, with the aim to list all the information needed to build the MBS classifier. The network is composed by three modules: the convolutional, the recurrent and the classification one, as shown in Figure S3. The model is fed with a sequence, representing a given metalloprotein structure, which is encoded as an array of size d x L, where L is the length of the sequence, d is the number of features representing the individual residues of the chain (29 here). The output of the convolutional module is still a sequence, having size d' x L, where d' is given by the architectural choice; d' is 29 as well. The output of the recurrent module is an array of size 15, which is then fed to the classification module; the latter gives the final classification as a size 2 array.

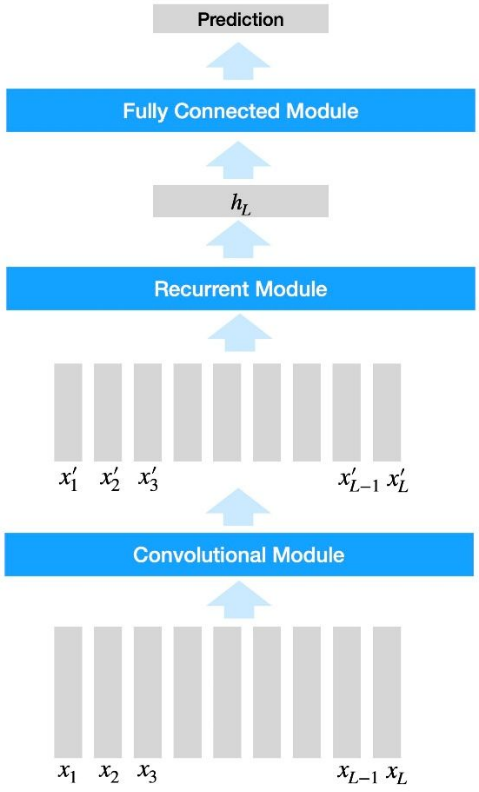

Figure S3. Architecture of the neural network. The arrows indicate how signals flow through the network.

Convolutional module

This module is implemented with two blocks as shown in Fig. S4. Each block is composed of a one-dimensional convolutional layer (conv1D), a batch-normalization operation (BN), a dropout regularization step and a ReLU activation function, Signals flow through the two blocks sequentially, as in the classical deep learning scenarios. The conv1D filter has a number of input channels equal to the number on input features, that is 29, and 29 output channels, architectural choice, with stride 1, padding 2 and kernel size 15. The dropout probability is 0.2. The same hyper parameters are used for the second block.

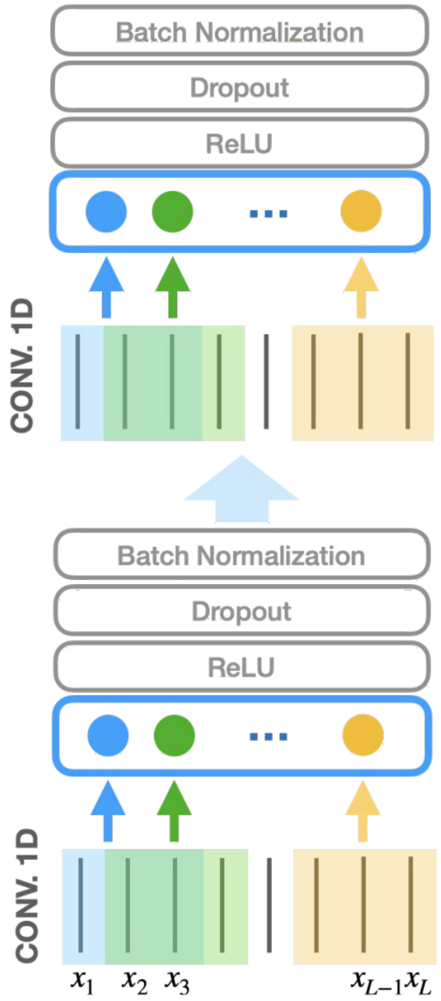

Figure S4. Architecture of the convolutional module

Recurrent Module

This module is implemented with a recurrent neural network (RNN) having Gated Recurrent Units (GRU) (Figure S5). The network has 3 layers each with 15 neurons, ReLU activation functions and dropout of 0.6. The input sequence is obtained from the convolutional module; therefore its shape is known. The RNN input size is constrained to be equal to the number of output channels in the second conv1D block.

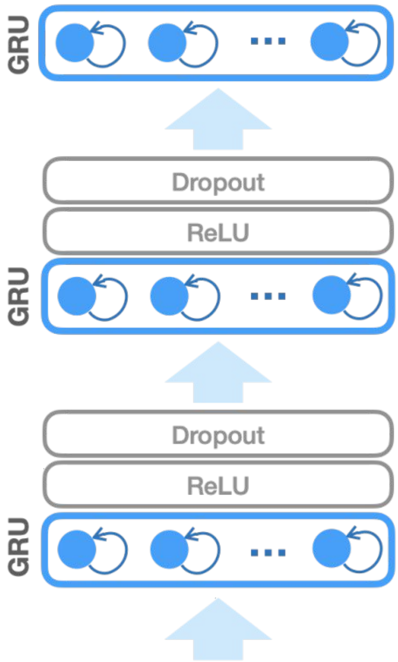

Figure S5. Architecture of the recurrent module

An RNN fed with a sequence generates a sequence as output. Anyway, here we only take the last element of the RNN-generated sequence to be processed by the subsequent module. Therefore, the output array has size 15.

Fully Connected Module

Once the array/embedding representing the site is generated by the RNN, it is fed in a fully connected layer (also known as linear layer), having a number of input units equal to the number of output units of the RNN (architectural constraint), that is 15, and 2 output neurons. These 2 neurons fire the class-probability distribution for the give input site fed as input to the model.

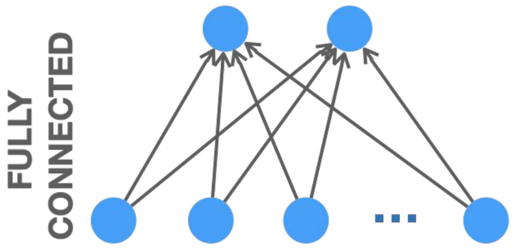

Figure S6. Architecture of the fully connected module

Training process

The zinc(II) dataset was randomly split in 10 stratified folds; accordingly, 10 different models have been trained, each with a different train-validation-test set, acting a classical cross-validation process.

Architecture parameters (such as the number of layers, dropout values etc.) were adjusted during the model search process, performed on a random subsample of the training data of a randomly chosen fold (to reduce computational time) and tested on its associated validation data The training process has been performed using the Adam optimization algorithm, (a variant of the classical stochastic gradient descent), with a learning rate 0.001, processing batch of 16 sequences per time, data is shuffled after each epoch. The training step is repeated for 200 epochs (Figure S7), and the model configuration (weights value) having the best performances (accuracy and loss value) on the validation set is kept. In the calculation of the loss value, different weights are given to data points of the different classes, in order to not be biased by the class having more points. The cost function used was the mean squared error (MSE).

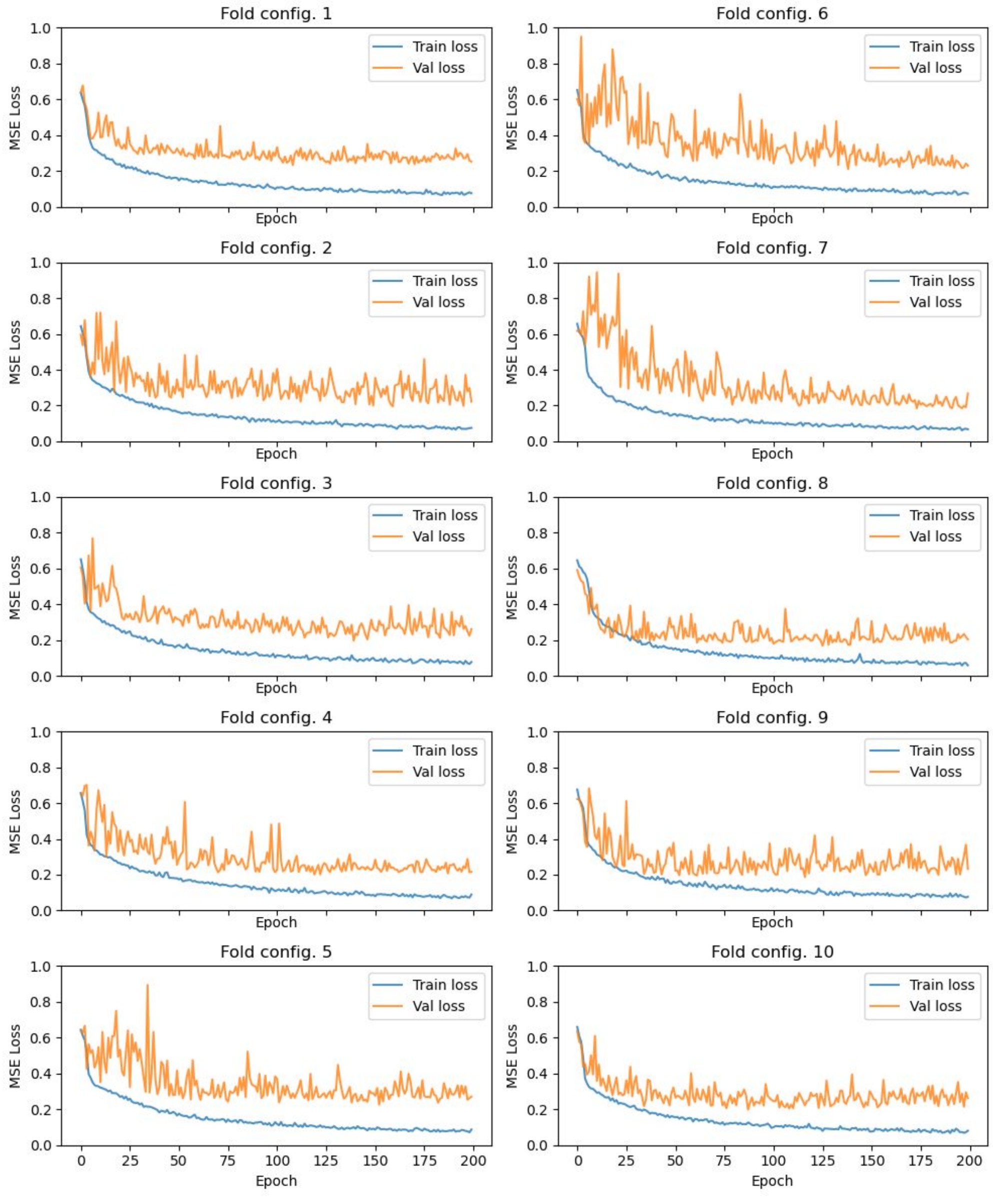

Figure S7. Learning curves of the individual folds (Val, orange: Validation; Train, blue: Training).

Software implementation

All the software has been implemented in Python 3, using the machine learning framework PyTorch. The scripts are available from the repository https://github.com/cerm-cirmmp/MBSDL

References

1. Lorimer, D. D.; Choi, R.; Abramov, A.; Nakazawa Hewitt, S.; Gardberg, A. S.; Van Voorhis, W. C.; Staker, B. L.; Myler, P. J.; Edwards, T. E., Structures of a histidine triad family protein from Entamoeba histolytica bound to sulfate, AMP and GMP. *Acta Crystallographica Section F* **2015,** *71* (5), 572-576.
2. Ye, J.; Kandegedara, A.; Martin, P.; Rosen, B. P., Crystal Structure of the *Staphylococcus aureus* pI258 CadC Cd(II)/Pb(II)/Zn(II)-Responsive Repressor. *Journal of Bacteriology* **2005,** *187* (12), 4214-4221.
3. Lefurgy, S. T.; Caselli, E.; Taracila, M. A.; Malashkevich, V. N.; Biju, B.; Papp-Wallace, K. M.; Bonanno, J. B.; Prati, F.; Almo, S. C.; Bonomo, R. A., Structures of FOX-4 Cephamycinase in Complex with Transition-State Analog Inhibitors. *Biomolecules* **2020,** *10* (5), 671.
4. Shouldice, S. R.; McRee, D. E.; Dougan, D. R.; Tari, L. W.; Schryvers, A. B., Novel Anion-independent Iron Coordination by Members of a Third Class of Bacterial Periplasmic Ferric Ion-binding Proteins*. *Journal of Biological Chemistry* **2005,** *280* (7), 5820-5827.
